# Interview with State Health Officer Brumby

**Published:** 1907-07

**Authors:** 


### INTERVIEW WITH STATE HEALTH OFFICER BRUMBY

POLICY OF THE GOVERNMENT WITH REFERENCE TO TUBERCULOSIS.

I called on Dr. Brumby and asked him what he was going to do about and with the indigent consumptives daily arriving in Texas.

He said:

Texas will debar all consumptives in the advanced stages from entering her confines. I am very desirous that the public should have a clear understanding of the conditions in Texas which makes such a step imperative.

In the first place, such a large tide of indigent consumptives has been pouring into the State that the people of certain sections are seriously burdened with such patients, many of whom have been in the State only a short while and have never done an hour's work since crossing the State line. A few dozen of these paupers could be easily cared for, but when they flock into Texas year in and year out it works a decided hardship on the taxpayers.

But there is another and far more important aspect to the question, and that is our inability to control the spread of the disease. The vast majority of these poverty-stricken unfortunates are without any means of livelihood and depend almost entirely upon charity for support. They are forced, therefore, to reside in populous communities—this results in their remaining collected in the cities where they do themselves least good and the public most harm.

If the poor unfortunates could reside out in the rural districts and were able to support themselves they could at least die in peace; but at present they are buffeted about the streets and suffer hardships that would undermine a strong man's constitution. It is true that our cities are at present caring for many tubercular patients, but the burden is more than they can properly or justly bear, and both the residents and itinerant consumptives suffer.

The public health is menaced all the more by reason of the fact that our State Health Department has not the means of sustenance for the individual, the authority to regulate their movements or to see that they take proper precautions to protect the public health. It is our hope that the next Legislature will make suitable changes in this direction so as to enable us to place some restrictions on the spread of the "White Plague." We are also hoping to see the Legislature provide a State sanitarium for the tubercular. But, in the meantime, we must call a halt on the armies of invalids that are beguiled into leaving their friends and homes by the vain delusion that they can come penniless into Texas and be restored to health. More of this class are already with us than we can care for. It seems imperative, then, that we should not receive others.

It is true the measure seems hard, but the real harshness has been with these well-meaning but short-sighted philanthropists who have encouraged many a man in the advanced stages of consumption to forsake home and loved ones and come into a strange land to die. The proper time for the afflicted to seek a better climate is before the disease has sapped their strength, and it is not humane to encourage them in the delusion that we have a fountain of health in Texas. It is my earnest wish that the world will think seriously on this question before pronouncing judgment.

